# Access and utilization of maternal healthcare in a rural district in the forest belt of Ghana

**DOI:** 10.1186/s12884-018-2159-5

**Published:** 2019-01-07

**Authors:** Gladys Buruwaa Nuamah, Peter Agyei-Baffour, Kofi Akohene Mensah, Daniel Boateng, Dan Yedu Quansah, Dominic Dobin, Kwasi Addai-Donkor

**Affiliations:** 1 School of Public Health, Kwame Nkrumah University of Science and Technology, Kumasi, Ghana; 2 Department of Biomedical Sciences, University of Cape Coast, Cape Coast, Ghana; 3 Amansie West District Health Directorate, Ghana Health Service, Manso Nkwanta, Ghana; 4 Ghana Health Service, Accra, Ghana

**Keywords:** Maternal healthcare, Antenatal care, Postnatal care, Skilled delivery, Limited-resource setting, Rural Ghana, Amansie west district

## Abstract

**Background:**

Poor maternal health delivery in developing countries results in more than half a million maternal deaths during pregnancy, childbirth or within a few weeks of delivery. This is partly due to unavailability and low utilization of maternal healthcare services in limited-resource settings. The aim of this study was to investigate the access and utilization of maternal healthcare in Amansie-West district in the Ashanti Region of Ghana.

**Methods:**

An analytical cross-sectional study, involving 720 pregnant women systematically sampled from antenatal clinics in five sub-districts was conducted from February to May 2015 in the Amansie-West district. Data on participants’ socio-economic characteristics, knowledge level and access and utilization of maternal health care services were collected with a structured questionnaire. Odds ratios were estimated to describe the association between explanatory variables and maternal healthcare using generalized estimating equations (GEE).

**Results:**

68.5, 83.6 and 33.6% of the women had > 3 antenatal care visits, utilized skilled delivery and postnatal care services respectively. The mothers’ knowledge level of pregnancy emergencies and newborn danger signs was low. Socio-economic characteristics and healthcare access influenced the utilization of maternal healthcare. Compared to the lowest wealth quintile, being in the highest wealth quintile was associated with higher odds of receiving postnatal care (adjusted odds ratio [aOR]; 95%CI: 2.84; 1.63, 4.94). Use of health facility as a main source of healthcare was also associated with higher odds of antenatal care and skilled delivery.

**Conclusion:**

This study demonstrates suboptimal access and utilization of maternal healthcare in rural districts of Ghana, which are influenced by socio-economic characteristics of pregnant mothers. This suggests the need for tailored intervention to improve maternal healthcare utilization for mothers in this and other similar settings.

**Electronic supplementary material:**

The online version of this article (10.1186/s12884-018-2159-5) contains supplementary material, which is available to authorized users.

## Introduction

Improved maternal health is an important pre-requisite for women’s advancement, yet due to low access and utilization of maternal healthcare, women, especially those in rural communities remain vulnerable and underserved. It is evident that the past decade has observed a dramatic improvement in the health of mothers, owing to improved maternal and childcare, nutritional practices and increased availability of low-cost and high impact public health measures such as Oral Rehydration Therapy (ORT) and vaccine for mothers/children [1]. According to the world health organization (WHO), specific interventions such as iron or folic supplementation for pregnant and postpartum women, vitamin A supplementation for children and postpartum women, malaria prophylaxis intervention such as insecticide-treated nets (ITNs), as well as Intermittent Preventive Treatment in pregnancy (IPTp) and dietary supplementation for pregnant or lactating mothers, have helped improve maternal and child healthcare [2]. In spite of these developments, more than half a million women die during pregnancy or childbirth or within a few weeks of delivery, with most of them living in developing countries [3]. These have been partly attributed to the low utilization of maternal healthcare services, and are also influenced by social, economic, and cultural factors as well as healthcare availability and accessibility [4–9]. In limited-resource settings, pregnant women do not receive the full benefits of maternal health services, with the benefits waning towards the rural and deprived communities.

We define maternal healthcare for the purpose of this manuscript to include antenatal care (ANC), skilled birth attendance and postnatal care (PNC). Focused ANC has been found to offer the opportunity for early detection and timely treatment of diseases, leading to improved maternal health outcomes. The detection and treatment of high blood pressure, for example, help to prevent eclampsia, and greatly reduce mortality [10]. Similarly, improved maternal outcomes have been observed through the detection and treatment of anemia [11]. The attendance of ANC is known to help augment healthcare during pregnancy through the provision of preventive health services, such as prophylactic treatment of malaria, the immunization against neonatal tetanus [12] and screening for sexually transmitted diseases such as HIV infection and hepatitis.

The assistance by a skilled birth attendant at delivery is also an important aspect of maternal care. Several babies or mothers are lost due to critical issues such as the inability to recognize delivery complications and ensuring quick referrals. Skilled delivery encompasses the presence of professionals (midwives, doctors, nurses, and others) during delivery. It also includes an enabling environment where the equipment, drugs and other supplies required for the effective and efficient management of obstetric complications are available [13]. The presence of skilled birth attendants (SBAs) in the community may help to reduce maternal mortality [14, 15] and this is regarded as, probably, one of the most critical interventions for reducing pregnancy-related deaths and disabilities in developing countries [13]. Report from the WHO shows a 50% reduction of maternal mortality in Egypt through the doubling of the proportion of births assisted by skilled professionals [16]. However, the provision of skilled attendance during delivery is only possible in the presence of functioning health systems [13], which include adequately trained and motivated workers, well-equipped facilities, transportation, and rapid referral systems. These factors are underdeveloped, inadequate or nonresistant in most developing countries’ health systems [14].

The postpartum period, which is usually 42 days after childbirth, is equally important for mothers. More than 60% of maternal deaths are known to occur during this period [17]. The death of a mother further exposes her newborn child to high risks of morbidity and mortality. In developing countries, the most common causes of maternal deaths during the postpartum period are hemorrhage, infections and hypertensive disorders [17]. These conditions and any other life-threatening or debilitating conditions that may require urgent medical attention could be identified during PNC. Other services and information, such as maternal and child nutrition, immunization, hygiene, and sanitation can all be provided during PNC. It is however reported that less than 30% of women in developing countries receive PNC [18].

Ghana has seen an improvement in the utilization of maternal health services over the years [19]. There has been an increase in deliveries in health facilities from 57% in 2008 to 73% in 2014 with an increasing number of ANC visits [19, 20]. These improvements are however minimal in regions with more rural communities [19]. Moreover, there is a paucity of evidence on the specific factors that explain access and utilization of maternal healthcare in deprived and rural communities in Ghana. This paper presents an analysis of factors that influence access and utilization of maternal healthcare in one of the deprived districts in the Ashanti Region of Ghana, Amansie-West.

## Methods

### Study design and setting

The details of the methods of the study have been described elsewhere [21]. An analytical cross-sectional study was conducted from February to May 2015 in the Amansie-West district of Ghana. The district is one of the most deprived districts in the Ashanti region and uniformly rural. It had a population of 149,437 and an annual growth rate of 2.7% as of 2014. The health system in the district is very weak, low health staff-to-patients (1: 74); doctor to population (719); nurse to population (1:2, 767) and midwife to women in reproductive age (WIRA) (1:4528) [22] ratios.

### Sampling and sample size

The study population was defined as confirmed pregnant women from 4 to 9 months. Seven hundred and twenty (720) pregnant women were systematically sampled from the various ANCs. The sample size was calculated with recourse to Cochran [23]; $\mathrm{n}\kern0.5em =\kern0.5em \frac{{\mathrm{Z}}^2\kern0.5em \mathrm{p}\left(1\kern0.5em -\kern0.5em \mathrm{p}\right)}{{\mathrm{d}}^{2.}}$

Where; *n* = the sample size

*Z* = the number relating to the degree of confidence anticipated in the result; in this case 95% confidence interval (*Z = 1.96* which is the abscissa of the normal curve).

*p* = an estimate of the proportion of people falling into the group in which we are interested clients of health care, where q = 1-p

*d* = proportion of error we are prepared to accept (sampling error; 5% anticipated error).

*n* = 1.96 ^2^ × 0.50 (1–0.50) ÷ 0.04 ^2^

### 3.842  0.25 / 0.04^2^ = 600

Due to attrition and incomplete data, an extra 20% (120 women) was added leading to a total of 720 respondents.

The participating ANCs were selected from five of the 10 sub-districts in the district. The required respondents from selected health facilities were proportional to the size of total eligible population per community. The distribution of respondents according to the sub-district was Manso Nkwanta 120, Edubia 141, Agroyesum 114, Antoakrom 140 and Esuowin 205 (Additional file 1: Table S1). At the five selected sub-districts, systematic random sampling technique was employed to select respondents from ANCs of private and public hospitals and health centers. This was guided by the sampling interval, *K*, estimated as the required sample size divided by the total attendants per facility. During the visit hours, a first participant was identified and interviewed as the starting point and the *Kth* respondent is approached, starting the count at the selected starting participant. This was repeated until the required sample size was attained

### Data collection and analysis

All participants involved in the study signed an informed consent form after explaining the objectives of the study. Participants had the right to withdraw from the study at any point in time during the data collection process. Data on respondents’ socioeconomic characteristics, access, and utilization to maternal health care services were collected using structured questionnaire after checking for clarity, consistency, and acceptability by pretesting. Data entry and analysis were done with SPSS for Windows (version 22) [24].

The outcome variable was maternal healthcare during the previous pregnancy, defined as ANC visits, skilled delivery, and PNC during previous pregnancy. The explanatory variables were socio-economic characteristics (age, education, religion, marital status, employment status, number of children, household wealth), access to healthcare (valid health insurance, proximity to health facility), healthcare seeking behavior (breastfeeding, use of family planning, preference of healthcare) and knowledge about pregnancy and danger signs. We calculated household wealth index with recourse to the procedure adopted in the demographic and health study (DHS) [25], using a simplified data on a household’s ownership of selected assets, such as televisions, bicycles, and farmlands. Scores were assigned to assets by using a Principal Component Analysis (PCA) and then standardized before grouping into quartiles. Details of the study variables are shown in Table 1. Univariable associations were tested using Chi-squared test and student t-test for categorical and continuous or discrete variables respectively. The influence of the explanatory variables on the odds of antenatal care, skilled delivery and postnatal care was estimated using generalized estimating equations (GEE) [26]. This helped to address the possible correlations of data within clinic groups. All statistical tests were performed at a significance level of *p* < 0.05.

**Table 1:** Study variables

Variable	Description
Maternal healthcare utilization	
Antenatal care	Three or more antenatal care visits during previous pregnancy.
Skilled delivery	Previous childbirth attended by a skilled birth attendant
Postnatal or postpartum care	Postnatal care for mother and baby received from a trained Health Care Worker (HCW) within three days or more during the previous pregnancy
Mothers socioeconomic characteristics	
Age	Maternal age (years) at the time of survey data collection; < 24 years, 24–34, and > 34.
Educational level	Mothers highest level of educational attainment (no education, primary, middle/JHS, secondary or tertiary level education)
Marital status	Married, cohabiting, single. Single included widowed, divorced/separated and never married.
Employment status	Employed; Unemployed, student, other
Number of children	Number of living children mothers had had prior to data collection
Religion	Christian, Islam, and other (other mainly consisted of traditional African religion, no religion)
Household wealth	Wealth class status of household expressed in quartiles: first quartile (lowest), second, third, fourth (highest).
Perceived socio-economic status	Self-rated socio-economic status; rich, moderately rich, poor, very poor.
Access to healthcare	
Registered with NHIS	Registered, not registered
Proximity to health facility	Time (in minutes) spent to travel to the health facility (< 30, 30–60, > 60).
Health behaviour and practices	
Use of family planning	Used any family modern planning method at six weeks postpartum; yes or no
Preferred source of healthcare	Place healthcare is sort for when mother or new-born baby is sick; herbalist, drugs at home, health facility, chemist, other sources
Knowledge level	
Knowledge of pregnancy-related conditions	Mothers’ ability to identify clinical conditions related to pregnancies; Good [8–10]; moderate [5–8]; low (< 5)
Knowledge of danger signs	Mothers’ ability to identify danger signs for new-born baby; Good [8–10]; moderate [5–8]; low (< 5)
Breastfeeding	Initiation of breastfeeding within 24 h after birth
Tetanus toxoid vaccination	Have had active immunization against tetanus

*NHIS* National Health Insurance Scheme*, JHS* Junior High School

## Results

### Relationship between respondents’ background characteristics and maternal healthcare

Table 2 shows the relationship between respondents’ background information and maternal healthcare. The median age of the women was 25 years and a majority, 57.5%, was in the age range of 20 to 29 years. Most women had basic education (primary, Junior High School or middle school) and 16.8% had no formal education. More than 85% were Christians and 9% were Muslims. With respect to their marital status, 65.5% were cohabitating whereas 28.8% were married. Two hundred respondents, constituting 27.8% had no child whereas 19.6% had more than three children. About 97% had registered with the National Health Insurance Scheme (NHIS) and almost all of them with the exception of two were active members of the scheme. The health facility was quite proximal to more than half of the women, who spent less than 30 min to reach the health facility. The distribution of wealth was quite proportional among the women, with 21.4 and 27.3% distributed in the lowest and highest quartile respectively.

**Table 2:** Relationship between respondents’ background information and maternal healthcare

Variables	Frequency *N* = 720	Percentage	Antenatal care visits > 3 (*N* = 493)	*p*-value	Skilled delivery (*N* = 602)	*p*-value	Postnatal care (*N* = 242)	*p*-value
			%		%		%	
Age								
− < 24	319	44.3	71.5	< 0.001	60.8	0.411	30.2	< 0.001
− 24–34	316	43.9	88.8		64.0		46.6	
− 34 and above	85	11.9	92.8		70.3		53.6	
Highest educational level								
− None	121	16.8	83.2	0.048	53.5	0.123	39.3	0.254
− Primary	148	20.6	86.2		65.7		46.3	
− Middle/JHS	357	49.6	81.9		67.0		40.1	
− Secondary/Tertiary/others	94	13.1	70.7		66.7		32.0	
Religion								
− Christianity	633	87.9	83.5	0.005	64.4	0.081	40.0	0.695
− Islamic	65	9.0	71.2		66.7		44.1	
− Other	22	3.1	61.1		33.3		33.3	
Marital status								
− Single*	55	7.7	81.9	< 0.001	66.9	0.485	47.4	0.032
− Married	208	28.9	85.3		61.8		38.5	
− Cohabitation	457	63.5	53.8		70.0		28.8	
Employment status								
− Unemployed/student/other	562	78.1	68.5	< 0.001	59.7	0.403	27.7	0.001
− Employed	158	21.9	85.8		64.9		43.9	
Number of living children								
− None	200	27.8	50.6	< 0.001	30.6	< 0.001	5.5	< 0.001
− 1	160	22.2	96.3		75.2		60.4	
− 2	118	16.4	92.5		63.4		49.7	
− 3 or more	342	33.6	91.5		61.6		50.4	
Registered with NHIS								
− Yes	697	97.1	88.9	0.426	76.9	0.318	40.5	0.543
− No	23	2.9	81.5		63.4		33.3	
Card active (*n* = 697)	695	99.7						
Proximity to health facility (minutes spent) (*n* = 719)								
− < 30	373	51.9	82.9	0.665	60.6	0.270	40.3	0.881
− 30–60	304	42.3	80.0		68.1		40.4	
− > 60	42	5.8	80.6		63.3		36.1	
Household wealth (*n* = 641)								
− First quartile (lowest)	137	21.4	85.7	0.031	61.1	0.598	29.5	< 0.001
− Second quartile	187	29.2	76.9		61.2		36.1	
− Third quartile	142	22.1	81.8		64.5		40.5	
− Fourth quartile (Highest)	175	27.3	89.0		68.5		56.1	
Perceived socio-economic status								
− Rich	46	6.4	92.5	< 0.001	88.6	0.014	65.0	0.010
− Moderately rich	486	67.5	85.5		62.8		37.7	
− Poor	143	19.9	70.5		58.8		40.2	
− Very poor	45	6.3	71.1		60.7		40.0	

*Include those divorced

The socio-economic characteristics of the mothers significantly influenced maternal healthcare services. ANC utilization, as well as access to PNC, increased with increasing age. The educational level (*p* = 0.048) and religious affiliation (*p* = 0.005) also had significant associations with ANC. Statistically, significant difference was also observed between the employed and unemployed with respect to utilization of ANC (*p* < 0.001) and access to PNC services (*p* = 0.001), with those employed being more likely to utilize these services. The proportion of mothers who optimally utilized ANC or either utilized skilled delivery and PNC differed significantly by the number of children a mother had.

### Respondents’ healthcare behavior and knowledge related to pregnancies and childcare

As shown in Table 3, most respondents breastfed their babies within 24 h of delivery. About 43% of the women used modern contraceptives at six weeks postpartum and the majority (53.5%) sought healthcare from health facilities for their newborn whereas 11.5% consulted the chemist (community medicine seller).

**Table 3:** Healthcare behaviors and practices related to pregnancy and childcare

Variables	Frequency	Percentage
Breastfed within 24 h after delivery (*n* = 629)		
− Yes	447	71.1
− No	182	28.9
Used family planning (any modern methods) at six weeks postpartum (*n* = 630)		
− Yes	271	43.0
− No	359	57.0
Preferred source of healthcare (n = 630)		
− Herbalist	6	1.0
− Drugs at home	38	6.0
− Health facility	337	53.5
− Chemist	71	11.5
− Prayer camps	35	5.6
− Others	143	22.7

**Fig. 1:**
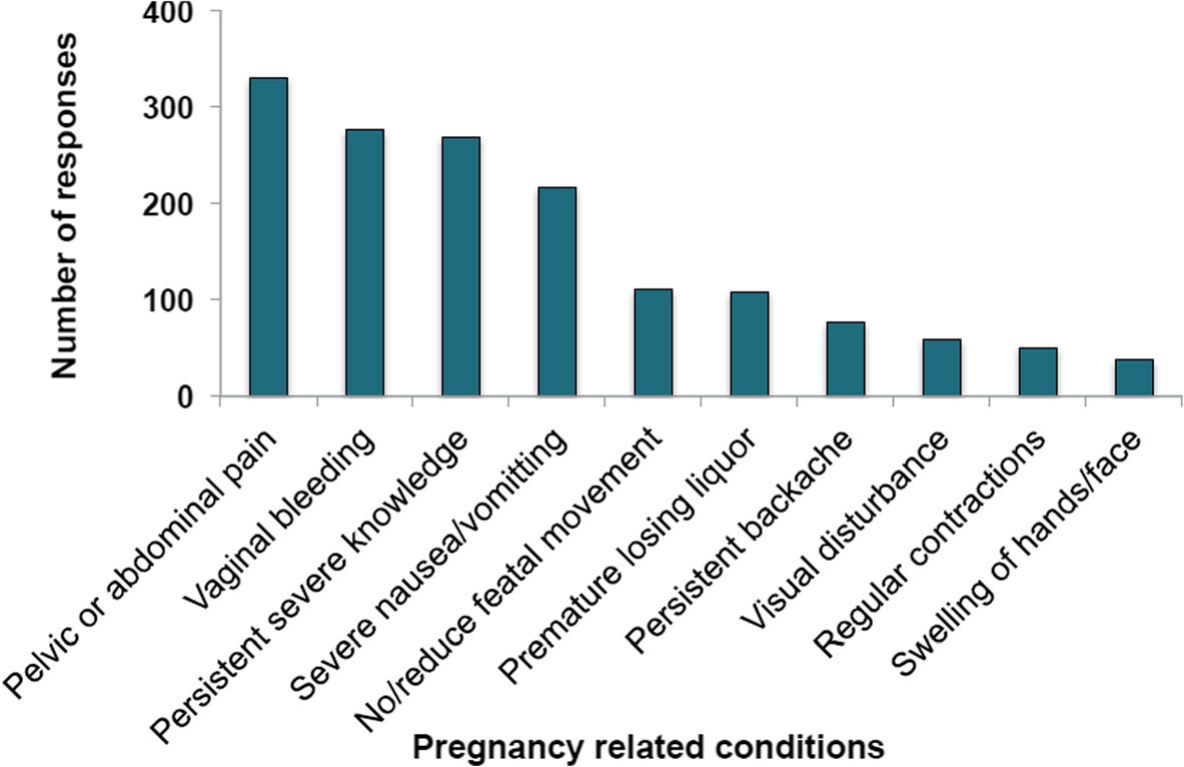
Respondents’ knowledge about pregnancy-related conditions among mothers in rural Ghana

**Fig. 2:**
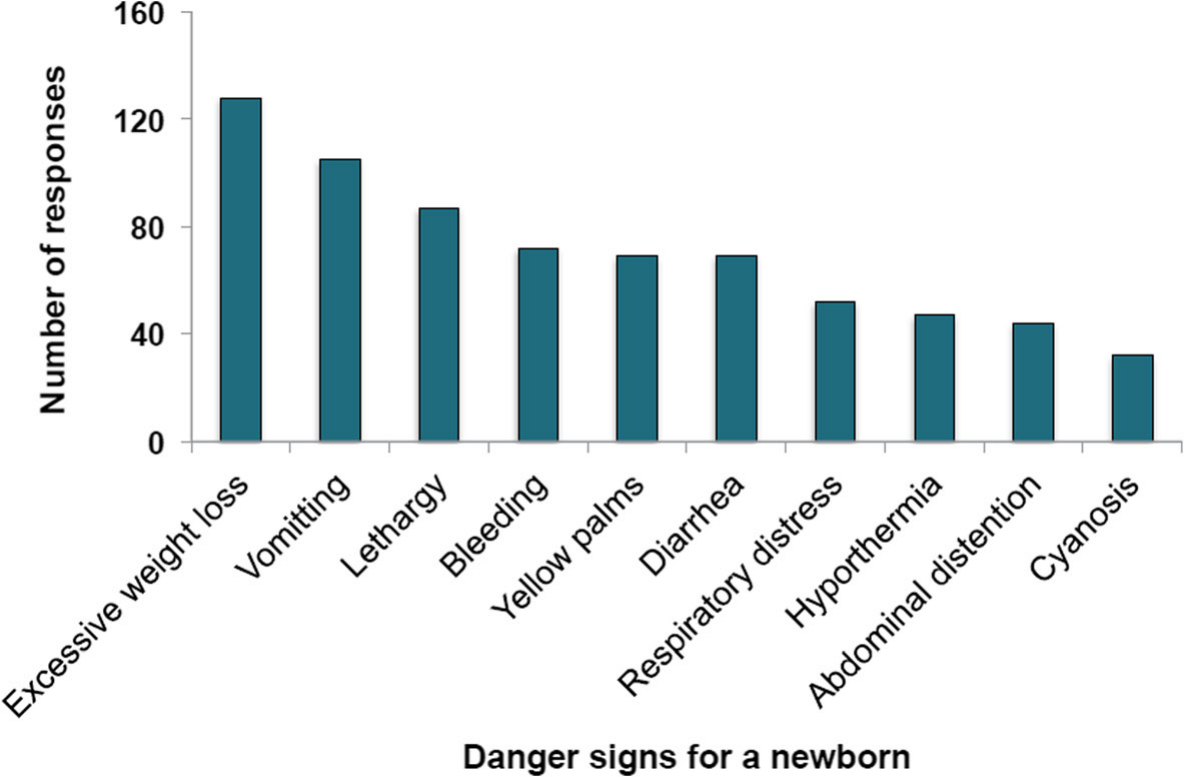
Knowledge of danger signs for a newborn among mothers in rural Ghana

Figures 1 and 2 show respondents’ knowledge on pregnancy related health conditions and danger signs for newborn babies respectively. Pelvic or abdominal pain was the most cited pregnancy related condition followed by vaginal bleeding. Regular contractions and swelling of hands or face were the least known among women in this study. As shown in Fig. 2, 128 women believed that excessive weight loss poses danger to the newborn baby. Other danger signs mentioned included lethargy, diarrhea, and respiratory distress with cyanosis being the least mentioned. Overall knowledge level was low among the respondents with 98.8 and 91.8% having low knowledge of danger signs of newborn and pregnancy-related conditions respectively.

Most (68.5%) women had three [3] or more antenatal visits during their previous pregnancies whereas 14.7% had none, Table 4. About 76% had postpartum care within three [3] or more. About 20% received no toxoid vaccines during previous pregnancies whereas 24.7 and 20.7% received one [1] and two [2] respectively. 33.6% of respondents received a day of postpartum care by a trained health care worker (HCW) whereas 20.6% were visited three days or more during postpartum by trained HCW. Only 20.3% received three [3] or more days of care for their babies by a trained HCW.

**Table 4:** Antenatal and postpartum care by healthcare workers

Variables	Frequency N = 720	Percentage
Number of antenatal visits during the last pregnancy		
− None	89	12.4
− 1	8	1.1
− 2	14	1.9
− 3 or more	493	68.5
− Unknown	116	16.1
Mean (SD)	5 (2.3)	
Last childbirth attended by a skilled birth attendant	602	83.6
Number of tetanus toxoid vaccines received during previous pregnancy		
− None	145	20.1
− 1	178	24.7
− 2	149	20.7
− 3 or more	248	34.4
Median	2.0	
Number of postnatal care received from a trained Health Care Worker (HCW) during previous pregnancy		
− 1	38	5.3
− 2	110	15.3
− 3 or more	456	63.3
− Unknown	116	16.1
Mean (SD)	4 (1.9)	
Care (at least within three days) of postpartum, by a trained HCW during previous pregnancy		
− None	118	16.4
− 1	242	33.6
− 2	212	29.4
− 3 times or more	148	20.6
Median	2.0	
Baby/ies receive care at least within three days after birth, by a trained HCW		
− None	118	16.4
− 1	240	33.3
− 2	215	29.9
− 3 times or more	146	20.3
Median	2.0	

### Factors associated with access to maternal healthcare services

Marital status was associated with their utilization of ANC, with mothers who were cohabiting having higher odds of attending ANC compared to those who were married, Table 5. Having the health facility as the usual source of care was also associated with higher odds of utilizing ANC compared to having other sources of healthcare.

**Table 5:** Logistic regression results of predictors of maternal healthcare utilization

Variables	Antenatal care visits > 3 aOR [95% CI]	Skilled delivery aOR [95% CI]	Postnatal care aOR [95% CI]
A. Socio-economic			
Age (ref = < 24)			
− 24–34	1.48 [0.77, 2.84]	1.64 [0.90, 3.01]	1.63 [0.97, 2.73]
− > 34	2.33 [0.58, 9.47]	2.73 [1.13, 6.59]*	2.06 [0.92, 4.63]
Highest educational level (ref = none)			
− Primary	1.34 [0.53, 3.39]	2.11 [1.10, 4.05]*	1.75 [0.97, 3.17]
− Middle/JHS	1.05 [0.49, 2.26]	2.18 [1.23, 3.87]**	1.40 [0.82, 2.40]
− Secondary/Tertiary/others	0.54 [0.22, 1.32]	1.39 [0.56, 3.46]	0.85 [0.40, 1.80]
Religion (ref = Christian)			
− Muslim	0.51 [0.22, 1.20]	1.52 [0.73, 3.15]	1.19 [0.63, 2.23]
− Other	0.30 [0.07, 1.27]	0.45 [0.10, 2.03]	1.11 [0.30, 4.08]
Employed	1.47 [0.77, 2.83]	0.76 [0.38, 1.55]	1.21 [0.69, 2.14]
Marital status (ref = married)			
− Cohabitation	2.07 [1.14, 3.77]*	0.92 [0.57, 1.48]	0.97 [0.63, 1.49]
− Single/Divorced	0.93 [0.31, 2.79]	2.42 [0.46, 12.84]	1.02 [0.38, 2.79]
Number of children (ref = 1 and 2)			
− 3 or more	2.15 [0.97, 4.77]	0.55 [0.31, 0.98]*	0.83 [0.49, 1.40]
Household wealth (ref = first)			
− Second quintile	0.73 [0.40, 1.50]	1.00 [0.53, 1.91]	1.45 [0.84, 2.49]
− Third quintile	0.71 [0.31, 1.63]	1.12 [0.58, 2.19]	1.63 [0.93, 2.86]
− Fourth quintile (Highest)	1.52 [0.63, 3.70]	1.39 [0.71, 2.70]	2.84 [1.63, 4.94]***
B. Access to healthcare			
Active NHIS membership	0.24 [0.04, 1.64]	0.47 [0.12, 1.86]	1.71 [0.57, 5.13]
Proximity to health facility (ref = < 30)			
− 30–60	0.61 [0.36, 1.03]	1.55 [0.99, 2.43]	0.95 [0.65, 1.39]
− > 60	0.49 [0.17, 1.41]	1.30 [0.50, 3.38]	0.78 [0.35, 1.75]
Health facility main source of care	3.45 [1.97, 6.03]***	2.36 [1.49, 3.73]***	0.83 [0.57, 1.20]
C. Knowledge level			
Knowledge about pregnancy related emergencies (ref = low)			
− Moderate	1.14 [0.44, 2.97)	0.88 [0.44, 1.75]	N/A
Knowledge about danger signs of newborn (ref = low)			
− High	0.47 [0.06, 3.67]	0.15 [0.01, 2.55]	0.33 [0.03, 3.81]

*p < 0.05; **p < 0.01;***p < 0.001; aOR = adjusted Odds Ratio

Access to skilled delivery was also associated with age, educational background, number of children and household wealth of the mother. Older mothers (> 34 years) had higher odds of skilled delivery compared to younger mothers (< 24 years) (OR; 95% CI: 2.7; 1.13, 6.59). Having some form of education was associated with skilled delivery as compared to no form of education. However, in contrast with access to ANC, having more children was associated with lower odds of having skilled delivery. Use of health facility as the main source of healthcare was also associated with higher odds of having skilled delivery (OR; 95% CI: 2.3; 1.49–3.73). Compared to the lowest wealth quartile, being in the highest wealth quartile was associated with higher odds of receiving postnatal care (OR; 95%CI: 2.66; 1.63, 4.94), Table 5.

## Discussion

This study sought to investigate access and utilization of maternal healthcare in five selected sub-districts in a rural district of Ghana. We found that mothers’ knowledge level of pregnancy emergencies and newborn danger signs were low. Mothers’ socio-economic characteristics and healthcare access were associated with their utilization of maternal healthcare.

### Respondents’ healthcare behavior and knowledge related to pregnancies and childcare

Most pregnant women in this study knew of pelvic or abdominal pain and vaginal bleeding as a pregnancy-related conditions. This is consistent with a study in Ethiopia, where about half of the study participants mentioned vaginal bleeding as a danger sign during pregnancy [27]. Other previous studies in similar settings have also reported on vaginal bleeding as the most cited danger sign during labor and in the postpartum period [28–30]. Previous evidence suggests that the major causes of maternal mortality are hemorrhage, sepsis, and hypertensive disorder during pregnancy. Adequate knowledge of these signs during pregnancy is necessary to ensure early detection and prompt consultation with the relevant health care professionals [31].

The women in this study also had some form of knowledge on conditions that could pose danger to the newborn baby. Weight loss was the most cited danger sign to the newborn baby while lethargy, diarrhea, respiratory distress, and cyanosis were least mentioned. The overall knowledge level was equally low with more than 90% unable to identify more than four danger signs. This corroborates previous findings of low knowledge of pregnancy-related danger signs [27]. In rural Uganda [32], only a fraction of pregnant women was found to have knowledge of one or more key danger signs in pregnancy. Enhancing women’s knowledge on obstetric care is essential to improving maternal healthcare services among pregnant women. Pregnant women’s knowledge about the need for ANC visit increases healthcare uptake, resulting in improved birth outcomes [33].

Undoubtedly, free maternal healthcare increases access and utilization [34]. However, women’s unawareness of free maternal health service, especially among vulnerable groups negatively influences the utilization of healthcare [35]. In Ghana, there has been a steady increase in the number of facility-based deliveries from about 300,000 in 2007 to about 500,000 in 2011 since the inception of free maternal healthcare in 2008. The utilization rate of maternal healthcare services in 2011 was about 66% [36]. Similar improvement in maternal healthcare and improved health status of women during pregnancy and in the postnatal period has been observed in Nigeria with the introduction of free maternal healthcare [36].

We found that most respondents sought alternative healthcare before presenting at their preferred source of healthcare. Some of the pregnant women purchased medicines from the chemist while others sought healthcare from other sources. A study in a rural western community of Kenya, also found that a third (32.4%) of mothers purchased and administered drugs to their sick children without seeking medical attention [37]. Similarly, a study in the slums of Nairobi, Kenya found that most mothers resort to chemical shops as their first source of healthcare, and when the care moves out of the home, private health facilities are used more compared to public health facilities [38]. This could result in late presentation at the health facility resulting in complications and deaths.

A large proportion of maternal and neonatal deaths are known to occur during the first 48 h after delivery. Prompt PNC for both the mother and the child are crucial in addressing any delivery-related complications and to ensure the provision of very important information to mothers on how to care for herself and her baby [39]. In this study, only 40% of the participants received a day of postpartum care by a trained healthcare worker and less than 30% received care for three days or more. This is similar to report by the 2014 Ghana Demographic and Health Survey (GDHS), where 40% had access to PNC care for more than three days after delivery [19]. Women who deliver at home are even more likely to miss out on this service. It is recommended for women who deliver at home to seek postnatal care services within 24 h, and for subsequent visits (including those who deliver in a health facility) [19].

Tetanus immunization of pregnant women is considered an important maternal issue in developing countries, recognizing the fact that neonatal tetanus is one of the leading causes of neonatal deaths in developing countries. About 65.8% of pregnant women involved in this study received two or more tetanus toxoid vaccines during their previous pregnancies. The 2014 GDHS [19] also reported tetanus injection coverage of 57% during previous pregnancies. Although most pregnant women had three [3] or more antenatal visits during their previous pregnancies, about one-fourth had none. It is obvious, therefore, that some pregnant women would miss essential maternal health services including tetanus toxoid immunization.

### Factors associated with access and utilization of maternal healthcare

Maternal age was significantly associated with access to maternal healthcare with older mothers being more likely to have access. Although there is a lack of clear consensus on the influence of age on maternal healthcare, the outcome of this study corroborates some previous findings [4, 5, 40]. A review of 21 DHS data from 21 countries in sub-Saharan Africa (SSA) also revealed that teenage mothers have poor maternal care than older women [41]. This could be as a result of societal stigmatization associated with teenage pregnancy, especially in rural settings, which deter pregnant mothers from seeking health care at the health facility.

Having some form of education was associated with higher odds of accessing and using maternal healthcare compared to those with no formal education. Previous studies in limited-resource settings showed a strong and positive association between maternal education and utilization of maternal healthcare [5–8, 42]. In Swaziland, literate women were found to use antenatal care, institutional deliveries, and PNC than those with no education [4]. The suggestion that women who are highly educated are better able to comprehend the importance of receiving prenatal care and are also more likely to know where to get it, may partly account for the observed association. Education is an opportunity to empower women, and empowered women have greater confidence and capability to make a decision to use modern health services for themselves and for their children.

The positive relationship between income and utilization of healthcare services is well documented [5, 21]. We found higher odds of maternal healthcare utilization among women in the highest household wealth index quartiles, compared to those in the lowest quartile. It has been argued that women from poor families or with limited financial resources may have difficulties paying for the cost of healthcare [9]. Women from households with higher economic status have the power to afford healthcare and have greater exposure to accessing relevant information related to maternal and child health. Other studies also argue that although household income may be high, women were more likely to utilize maternal healthcare when they have personal control over finances [43], and therefore the interaction between household wealth and autonomy produces higher healthcare utilization [6].

### Limitations of the study

This study provides important evidence on the utilization of maternal healthcare in rural Ghana. By using the GEE to assess the association between the independent variables and utilization of maternal health services, we were able to deal with the clustering within clinic groups. The choice of cross-sectional study design, however, could not permit to make direct inferences on the relationship between the outcome(s) variable and the covariates studied. Recruiting only pregnant women presenting at ANC is inherent with selection bias, and could lead to overestimation of the level of utilization, as users of maternal healthcare services are more likely to have utilized it in the past. The level of utilization is however consistent with the level of maternal health utilization reported in the 2014 GDHS, ANC care 97%, skilled delivery 74% and postnatal care 74% [44].

## Conclusion

This study demonstrates suboptimal access and utilization of maternal healthcare in a rural district in Ghana, which was associated with socio-economic characteristics such as household wealth index, educational background, and number of children. Our study also documents very low knowledge of pregnancy-related emergencies and danger signs of newborn among mothers. Looking at the gaps in healthcare access and utilization, educational interventions that utilize both health facility and community-based educational approaches will be required to reach all mothers in this and similar rural settings.

## Additional file

Additional file 1: **Table S1.** Brief description of the data: The distribution of respondents according to the study communities or sub districts. (DOCX 17 kb)

## Abbreviations

ANC: Antenatal care
DHS: Demographic and health study
GEE: Generalized estimating equations
NHIS: National Health Insurance Scheme
ORT: Oral Rehydration Therapy
PCA: Principal Component Analysis
PNC: Postnatal care
SBAs: Skilled birth attendants
WHO: World Health Organization
WIRA: Women in reproductive age
