# Supplementary material for: Access and utilization of maternal healthcare in a rural district in the forest belt of Ghana
Source: BMC Pregnancy Childbirth. 2019 Jan 7;19:6. doi: 10.1186/s12884-018-2159-5 (PMC6322319; doi:10.1186/s12884-018-2159-5)
Supplement: Supplementary file 1 — Table S1. Brief description of the data: The distribution of respondents according to the study communities or sub districts. (DOCX 17 kb) [file 12884_2018_2159_MOESM1_ESM.docx]

**Supplementary Table 1:** Sample distribution from the study communities

| **Sub-district** | **Total population** | **4% WIRA** | **Sample size** |
| --- | --- | --- | --- |
| Antoakrom | 17706 | 708 | 140 |
| Agroyesum | 14, 428 | 577 | 114 |
| Manso Nkwanta | 15,187 | 607 | 120 |
| Edubia | 17,777 | 711 | 1401 |
| Esuonwin | 25,997 | 1040 | 205 |
|  |  | 3644 | 720 |

*WIRA, Women in reproductive age*
